# Physiological Changes of *Parachlorella Kessleri* TY02 in Lipid Accumulation under Nitrogen Stress

**DOI:** 10.3390/ijerph16071188

**Published:** 2019-04-02

**Authors:** Yifan Gao, Jia Feng, Junping Lv, Qi Liu, Fangru Nan, Xudong Liu, Shulian Xie

**Affiliations:** School of Life Science, Shanxi University, Taiyuan 030006, China; cherrygaoyifan@163.com (Y.G.); fengj@sxu.edu.cn (J.F.); lvjunping024@sxu.edu.cn (J.L.); liuqi@sxu.edu.cn (Q.L.); nanfr@sxu.edu.cn (F.N.); liuxudong@sxu.edu.cn (X.L.)

**Keywords:** *Parachlorella kessleri* TY02, nitrogen stress, lipid synthesis

## Abstract

In order to study the effects of nitrogen stress on the lipid synthesis of *Parachlorella kessleri* TY02 and to understand the changes in growth, photosynthetic pigments, total protein and total carbohydrate contents during lipid accumulation, the cells of the strain were cultured in nitrogen-deficient (N^−^) and nitrogen-rich (N^+^) media for one week. Changes in cell growth, chlorophyll content, chlorophyll fluorescence parameters, neutral lipid and total lipid content, total protein content and total carbohydrate content were measured and analyzed. The results showed that, under nitrogen stress, the algal strain grew slowly, and chlorophyll and total protein contents decreased, while total carbohydrate and total lipid contents increased. This indicated that, under nitrogen stress, most of the carbon flowed to the synthesis of lipids and carbohydrates. Meanwhile, reducing the nitrogen content was a relatively economical and easy to operate method of promoting lipid accumulation.

## 1. Introduction

With the rapid trend of modernization, more and more energy is needed. The accelerated consumption of petroleum, coal and other fossil energy gives rise to two major problems—energy shortage and environmental pollution. Therefore, it is urgent to develop clean renewable energy [1]. As an important phytoplankton, microalgae can provide many valuable bio-products such as lipids, pigments, bioactive compounds and certain polysaccharides, so they have the potential for successful market penetration [2]. Biodiesel is considered a good alternative to fossil energy due to its high combustion performance, wide application range, safety and reliability, as well as its being less detrimental to the environment. Especially, microalgae have many merits of their own, such as diverse species, wide distribution, fast growth, rich in oil and fat, and no occupation of cultivated land, which makes them ideal biodiesel raw materials [3,4,5]. At present, the research on microalgae biodiesel in China is still in the initial stage and the high harvesting cost and low oil yield are still two key factors hindering its development. The primary tasks of industrialized production include screening and cultivating high-lipid algal strains, as well as optimizing cultivation conditions [6]. Recently, several studies have reported that temperature, salts and light intensity are considered major environmental stressors to boost lipid accumulation in microalgae [7,8,9]. Besides, lipid improvement in microalgae also can be achieved through the modulation of cultivation conditions and/or by varying stress conditions, for example, Satpati et al. discovered that phosphorus deficiency could promote the synthesis of lipids in *Chlorella ellipsoidea* and *Chlorococcum infusionum* [10]. Another study reported that Concas et al. found that suitable concentrations of iron were beneficial to accumulating lipids in *Chlorella vulgaris* [11]. 

Nitrogen is a key element of many important life processes. It exists in the basic amino acid unit of proteins and is also one of the five basic elements of nucleic acids such as DNA and RNA. Additionally, nitrogen is one of the main elements of chlorophylls in plants. Consequently, nitrogen is one of the most consumed elements in microalgal cultures, especially in the early stages, and thus is the main nutrient affecting the growth and lipid content of microalgae. Research has shown that by removing nitrogen from the culture medium the local environment can be controlled, leading to nitrogen stress [12]. Under stress conditions and as growth enters the aging stage, the lipid content of microalgae—especially the neutral lipid content—increases [13]. However, there are only a few studies on the changes in protein and carbohydrate content during the accumulation of lipids in low nitrogen conditions [14,15,16].

In this study, a strain of high-lipid microalgae was cultured optimally for nitrogen deficiency, so as to preliminarily assess the changes in nutrient metabolites under nitrogen stress.

## 2. Materials and Methods

### 2.1. Algal Strain and Cultivation

The algal strain *Parachlorella kessleri* TY02 (Chlorophyta and Chlorellaceae), which was first isolated from soil from Shanxi Province in the north of China and described in Reference [17], was used in this study. 

### 2.2. Growth Conditions and Nitrogen Stress Treatment

*P. kessleri* TY02 was grown on Tris-acetate-phosphate (TAP) medium [18] with pH 7.0 ± 0.1. The nitrogen-sufficient (N^+^) medium used in this study was TAP medium, whereas the nitrogen-deficient (N^−^) medium was TAP medium with NaCl instead of NH_4_Cl. 

*P. kessleri* TY02 grown to logarithmic phase was inoculated into a 1-L Erlenmeyer flask containing 800 mL TAP medium at an initial concentration of about OD_687_ 0.2 (dry weight of about 0.07 g L^−1^). The mixture was then placed in a light incubator (BSG-300, Boxun, Shanghai, China) at 25 °C, 12 h light: 12 h dark, and 3000 lux.

After growing to the mid-logarithmic phase, algal cells were harvested by a centrifugal machine (5430R, Eppendorf, Hamburg, Germany) at 4 °C and 5000 rpm for 10 min. The supernatant was discarded. After being washed with N^−^ TAP medium for three times, the algal was used to inoculate the control group (N^+^ TAP medium) and the experimental group (N^−^TAP medium), respectively, and then cultured in 1-L Erlenmeyer flasks. The initial concentration was OD_687_ 0.2. All experiments were performed in triplicate and the culture conditions were the same as above for 7 d. The algal solution was shaken about three times a day. The measurements and analysis were performed around the same time every day.

### 2.3. Determination of Algal Growth

#### 2.3.1. Absorbance

The maximum absorption peak of *P. kessleri* TY02 was searched with an ultraviolet-visible (UV–Vis) spectrophotometer (TU-1810, Puxi, Beijing, China). The samples were scanned by full wave to determine the corresponding wavelength of the maximum absorption peak, with distilled water as blank control and the result was 687 nm. OD_687_ values of the N^+^ and N^−^ conditions were subsequently determined with distilled water as the blank control.

#### 2.3.2. Dry Weight

The dry weight of the microalgal biomass was determined according to Lv et al. [19]. Algal cultivation broth (5 mL) was filtered by pre-drying and weighing a membrane with the aperture of 0.45 μm, then dried to a constant weight and weighed. The difference in quality between the two values was recorded as the algal dry weight. The formula is as follows:

(1) $$\mathrm{DW}=\left(m2-m1\right)\;V^{-1}$$

where DW is the microalgal dry weight (g L^−1^), m1 (g) is the constant dry weight of the filter membrane, m2 (g) is the total weight of the filter membrane and algal solution after extraction and filtration, and V (L) is the volume of the algal cultivation broth in Equation (1).

### 2.4. Determination of Chlorophyll and Carotenoids

The contents of chlorophyll and carotenoids were determined according to Mera et al. [20]. First, 3 mL of algal cultivation broth were taken and centrifuged at 5000 rpm for 10 minutes, then the supernatant was discarded. Subsequently, 3 mL of 95% ethanol solution were added into the algal sludge and mixed evenly. The algal cell wall was broken by an ultrasonic breaker (SCIIENTZ-IID, Xinzhi, Ningbo, China) at 20% for 10 min. The mixture was wrapped in tin foil and put in a refrigerator (dark and low temperature environment) at 4 °C, for 24 h. Then, the solution was centrifuged at 5000 rpm for 10 min. The supernatant was saved for subsequent experiments. The OD_665_, OD_649_ and OD_470_ values were determined with 95% alcohol solution as the blank control. The contents (mg L^−1^) of chlorophyll a (Chl a) in Equation (2), chlorophyll b (Chl b in Equation (3), total chlorophyll (Chl (a + b)) in Equation (4), and carotenoids (Car) in Equation (5) were respectively calculated according to the following formulae:

(2) $$\mathrm{Chl}\;a=13.95\times {\mathrm{OD}}_{665}-6.88\times {\mathrm{OD}}_{649}$$

(3) $$\mathrm{Chl}\;b=24.96\times {\mathrm{OD}}_{649}-7.32\times {\mathrm{OD}}_{665}$$

(4) $$\mathrm{Chl}\left(a+b\right)=18.08\times {\mathrm{OD}}_{649}+6.63\times {\mathrm{OD}}_{665}$$

(5) $$\mathrm{Car}=\left(1000\;{\mathrm{OD}}_{470}-2.05\times \mathrm{Chl}\;a-114.8\times \mathrm{Chl}\;b\right)/245$$

### 2.5. Determination of Chlorophyll Fluorescence

After storing 3 mL algal cultivation broth in the dark for 20 min, three PSII indexes of chlorophyll fluorescence were determined according to Markou et al. [21] by the portable PAM fluorometer (AquaPen-C AC100, Prague, Czech). The three indexes included the maximum quantum yield of photosystem II photochemistry (Fv/Fm), the potential activity of photosystem II (Fv/F0) and the total light energy flux (PIABS values).

### 2.6. Determination of Neutral Lipid Content

Nile Red (NR) is a fat-soluble fluorescent dye. Neutral lipids can combine with NR to produce bright yellow fluorescence and changes in neutral lipid content can be determined by detecting the fluorescence intensity [22].

Algal cultivation broth samples were collected for determination every day. First, 240 μL algal cultivation broth were added to a 96-black well plate, then 1 μL (0.5 mg mL^−1^) NR (9-diethylamino-5H-benzo (a) phenoxazine-5-one, dissolved in DMSO) was rapidly added under the dark environment. After mixing, the plate was incubated for 10 min, at 37 °C, to allow the NR and neutral lipids to combine. The fluorescence intensity was determined by the Multiscan Spectrum (Epoch2, BioTek, VT, USA) and the excitation and emission wavelengths were 543 nm and 598 nm, respectively. A mixture of NR and culture medium was used as positive control, while algal cultivation broth without NR was used as negative control. The fluorescence intensity of the algal cultivation broth was calculated by removing the values of controls. The fluorescence intensity of the algal cultivation broth was calculated according to the following formula [23,24]:

(6) $$\mathrm{sFI}=\mathrm{FI}\times {\mathrm{BW}}^{-1}$$

where sFI is the specific fluorescence intensity of the per unit volume of the cell (a.u. mg^−1^), FI is the total NR fluorescence intensity of the algal cultivation broth (a.u. 240 μL^−1^), and BW is the biomass weight of 240 μL algal cultivation broth in Equation (6).

### 2.7. Confocal Laser Scanning Microscopy

After being stained with NR, the algal cells were scanned under a confocal laser microscope (LSM880, Zeiss, Oberkochen, Germany). Clear cells were selected to capture pictures of the optical sections with the strongest signals. The cell lipid content was reflected by the fluorescence intensity in the range of the excitation wavelength and fluorescence signal. The excitation wavelength of NR fluorescence was 488 nm, and the emission wavelength was 550-600 nm. The excitation wavelength of chlorophyll self-fluorescence was 630 nm, and the emission wavelength was 630-700 nm. The image processing system of the laser confocal microscope was used for image processing [25].

### 2.8. Determination of Total Lipid Content

About 0.1 g frozen dried algal powder were weighed and placed in a 5-mL glass bottle. Then, 3 mL methanol and 1.5 mL chloroform were added to the bottle. The solution was mixed on a vortex oscillator and the cell walls were then broken by an ultrasonic crusher (SCIENTZ-IID, Scientz, Ningbo, China) at 25% (at the rated power), for 10 min. Next, the mixture was centrifuged (5430R, Eppendorf, Hamburg, Germany) at 5000 rpm min^−1^ for 10 min. To the resulting supernatant, 1.5 mL 0.75% KCl aqueous solution and 1.5 mL chloroform solution were added, followed by shaking and mixing with the vortex oscillator. The mixture was then centrifuged at 5000 rpm min^−1^ for 10 min and the precipitate liquid was retained. After repeating the above steps twice and combining all the liquids in a 5-mL glass bottle, the mixture was blown dry with nitrogen and then weighed [26]. The total lipid content and lipid productivity were calculated according to the following formula:

(7) LC(%)=(w3−w2)/w1×100%

where LC (%) is the weight percentage of the total lipid in the algal powder, w1 (g) is the dry weight of the algal powder, w2 (g) is the weight of empty bottle, and w3 (g) is the total weight of the algal lipids and the glass bottle after the algal lipids were extracted in Equation (7).

(8) $$\mathrm{BP}\left({\mathrm{mg}\;L}^{-1}d^{-1}\right)=\mathrm{DW}/T\times 100$$

where BP (${\mathrm{mg}\;L}^{-1}d^{-1}$) is the biomass productivity of the alga, DW (g) is the dry weight of the algal powder, T (d) is the time of the cultivation were extracted in Equation (8).

(9) $$\mathrm{LP}\left({\mathrm{mg}\;L}^{-1}d^{-1}\right)=\mathrm{BP}\;\mathrm{LC}$$

where LP (${\mathrm{mg}\;L}^{-1}d^{-1}$) is the lipid productivity of the alga, BP (${\mathrm{mg}\;L}^{-1}d^{-1}$) is the biomass productivity of the alga, LC is the weight percentage of the total lipid in the algal powder were extracted in Equation (9).

### 2.9. Determination of Fatty Acid

The fatty acid content was analyzed with a gas chromatography-mass spectrometry instrument (GC-MS) (7890A-5975C, Agilent, Los Angeles, USA). For the GC-MS analysis, the column RTW-WAX (30 m × 0.25 mm, 0.5 μm) at a temperature program starting from 50 °C to 150 °C and kept for 2 min, raised to 200 °C with the speed of 10 °C min^−1^ and stained 6 min, raised to 230 °C with the speed of 10 °C min^−1^ for 30 min, raised to 240 °C with the speed of 10 °C min^−1^ for 10 min, was used. As carrier gas nitrogen at a flow velocity of 0.35 mL min^−1^ was used and electron ionization (EI) source at an electron energy of 70 eV. The mass spectrum scanning range was m z-1 20-450 and the sample size was 0.2 μL. A mass spectral data base NIST 05 was used according to the description by Liu et al. [27]. 

### 2.10. Determination of Total Protein Content

The algal cultivation broth was centrifuged at 5000 rpm min^−1^ for 10 min, and the supernatant was removed. Then, 0.5 M NaOH was added to the algal sludge and heated in a water bath at 90 °C for 1 h. The total protein content was determined according to He et al. [28].

### 2.11. Determination of Total Carbohydrate Content

After adding 6 M HCl, the algal cultivation broth was heated to 100 °C in a water bath for 30 min. Subsequently, the solution was cooled and 6 M NaOH was added for neutralization. The total crude carbohydrate was determined by the phenol sulfuric acid method described by Prajapati et al. [29].

### 2.12. Statistical Analysis

The experiment was replicated three times. All data were expressed as mean ± standard error. A completely randomized design was used. A t-test was used to determine the statistical significance of differences between mean values at *p* ≤ 0.05. All statistical analyses were carried out using the SPSS 19.0 statistical software (IBM Inc. Chicago, IL, USA).

## 3. Results

### 3.1. Algal Growth

**Figure 1:**
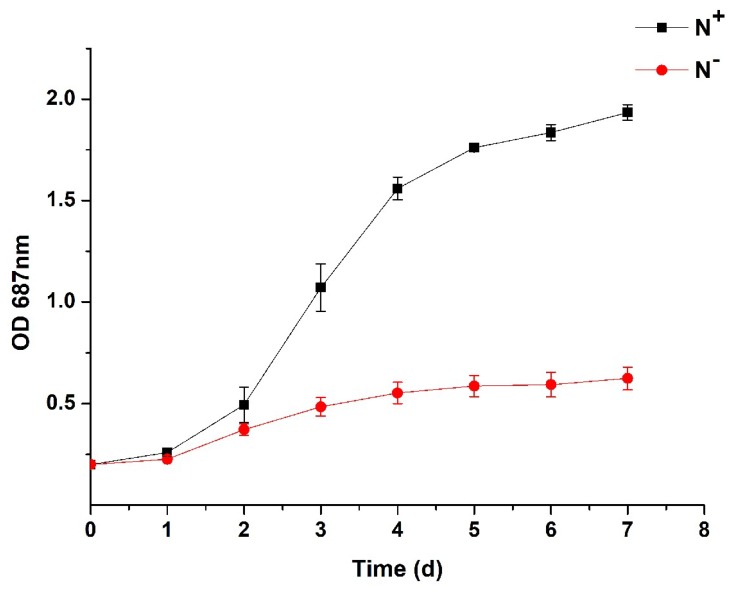
Absorbance comparison between N^+^ and N^−^ of *Parachlorella kessleri* TY02. Data are shown as mean ± SE. *n* = 3.

In the experimental cycle, the absorbance (OD_687_) of *P. kessleri* TY02 at both N^+^ and N^−^ showed the same upward trend but the algal growth was faster at N^+^ than at N^−^ (Figure 1). Initially, the OD_687_ under the two treatments was almost the same, with a value of about 0.4. After one day, the algal growth of both N^+^ and N^−^ entered the logarithmic phase but it was significantly faster at N^+^ than at N^−^. On the 7th day, the OD_687_ at N^+^ was as high as 1.9, while that at N^−^ was only about 0.6. Therefore, nitrogen deficiency slowed down the algal growth.

**Figure 2:**
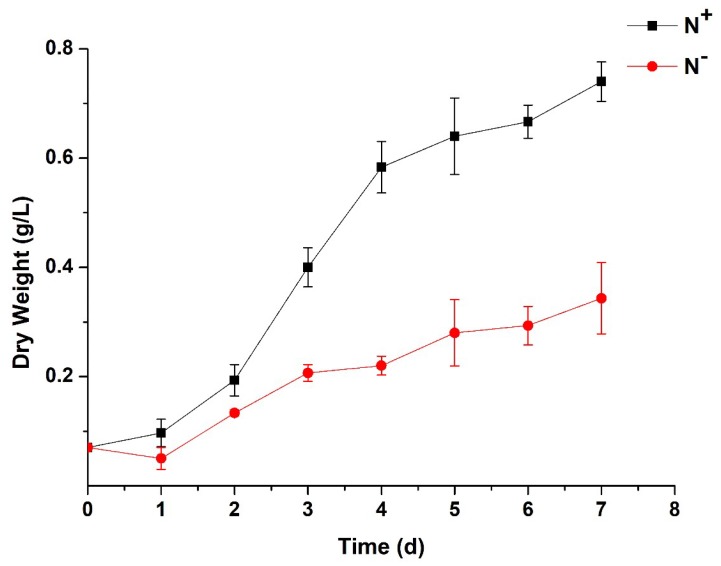
Dry weight comparison between *Parachlorella kessleri* TY02 grown under N^+^ and N^−^. Data are shown as mean ± SE. *n* = 3.

As shown in Figure 2, the change in algal dry weight at N^+^ shared the same trend with that at N^−^. In the experimental cycle, the algal dry weight of *P. kessleri* TY02 at both N^+^ and N^−^ showed a similar upward trend but the algal dry weight increased significantly faster at N^+^ than at N^−^. Initially, the algal dry weight under the two treatments was almost the same, with a value of about 0.07 g L^−1^. After one day, the algal growth under both N^+^ and N^−^ entered the logarithmic phase but it was significantly faster at N^+^ than at N^−^. On the 7th day, the algal dry weight at N^+^ was 0.74 g L^−1^, while that at N^−^ was only 0.34 g L^−1^. This result further illustrated that nitrogen deficiency can slow down the algal growth.

### 3.2. Contents of Chlorophyll and Carotenoids and Analysis of Chlorophyll Fluorescence

**Figure 3:**
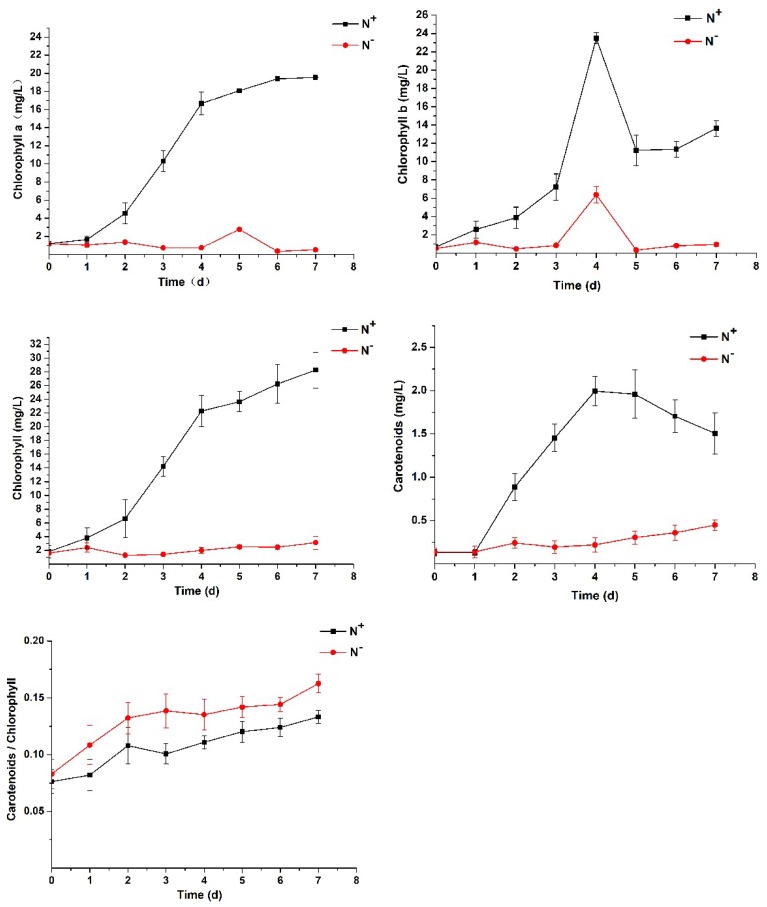
Comparison of chlorophyll and carotenoids contents of *Parachlorella kessleri* TY02 under N^+^ and N^−^ culture media. Data are shown as mean ± SE. *n* = 3.

The chlorophyll and carotenoids contents of *P. kessleri* TY02 are shown in Figure 3. The contents of Chl a, Chl b, Chl (a + b) and Car increased significantly under N^+^ medium. In contrast, under the N^−^ medium, these contents only increased slowly and the increase was not significant. On the 7th day, the contents of Chl a, Chl b, Chl (a + b) and Car under N^−^ were only 2.64%, 6.99%, 11.07% and 29.78% of those under N^+^, respectively. 

**Figure 4:**
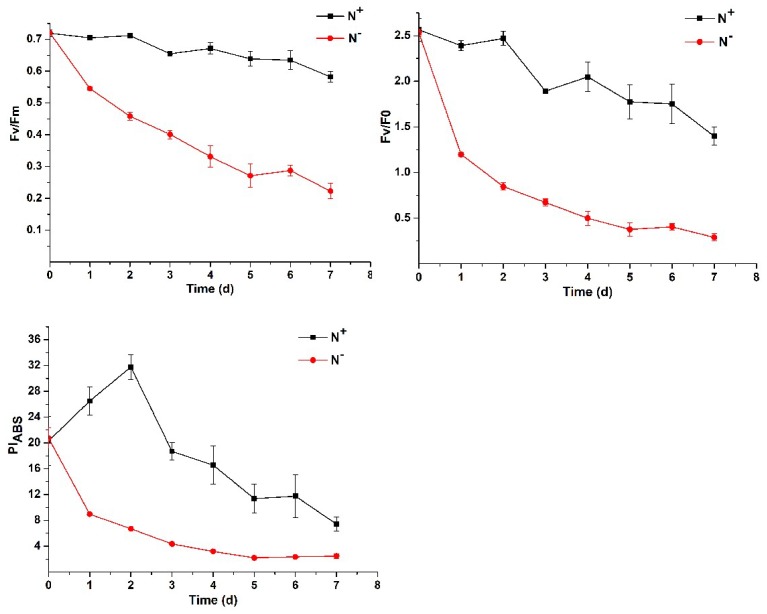
Comparison of chlorophyll fluorescence parameters of *Parachlorella kessleri* TY02 under N^+^ and N^−^ culture media. Data are shown as mean ± SE. *n* = 3.

As seen in Figure 4, the chlorophyll fluorescence parameters, Fv/Fm, Fv/F0 and PI_ABS_, showed a downward trend both under N^+^ and N^−^ but were significantly lower under N^−^ than under N^+^. On the 7th day, the values of Fv/Fm, Fv/F0 and PI_ABS_ under N^−^ decreased from 0.720, 2.532 and 20.749 to 0.223, 0.288 and 2.445, respectively; under N^+^, the three values decreased from 0.719, 2.564 and 20.294 to 0.582, 1.396 and 7.403, respectively. These results further indicate that nitrogen deficiency severely inhibits the photosynthetic capacity and PSII activity of *P. kessleri* TY02.

### 3.3. Analysis of Neutral Lipids Content

**Figure 5:**
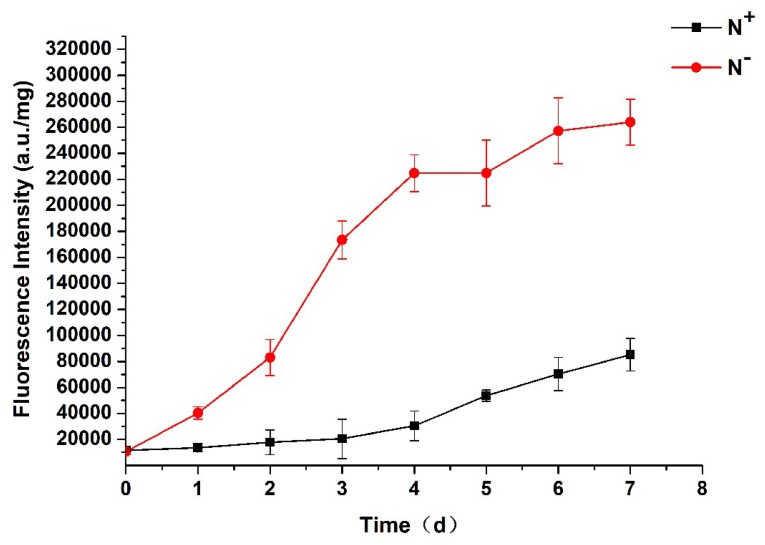
Comparison of neutral lipid contents of *Parachlorella kessleri* TY02 under N^+^ and N^−^ culture media. Data are shown as mean ± SE. *n* = 3.

The fluorescence intensity of algal cells stained with NR was significantly correlated with the content of neutral lipids in the cells. The algal neutral lipid contents can be evaluated by sFI, which represents the NR fluorescence intensity of the per unit volume of algal cells (a.u./mg). As shown in Figure 5, the sFI values under both N^−^ and N^+^ show an upward trend, indicating that the neutral lipids of *P. kessleri* TY02 accumulate continuously. By comparison, the content of neutral lipids under N^−^ medium was significantly higher than that under N^+^. On the 7th day, the sFI under N^−^ was 2.87 times that under N^+^. The results show that nitrogen stress was beneficial to the accumulation of neutral lipids in *P. kessleri* TY02.

**Figure 6:**
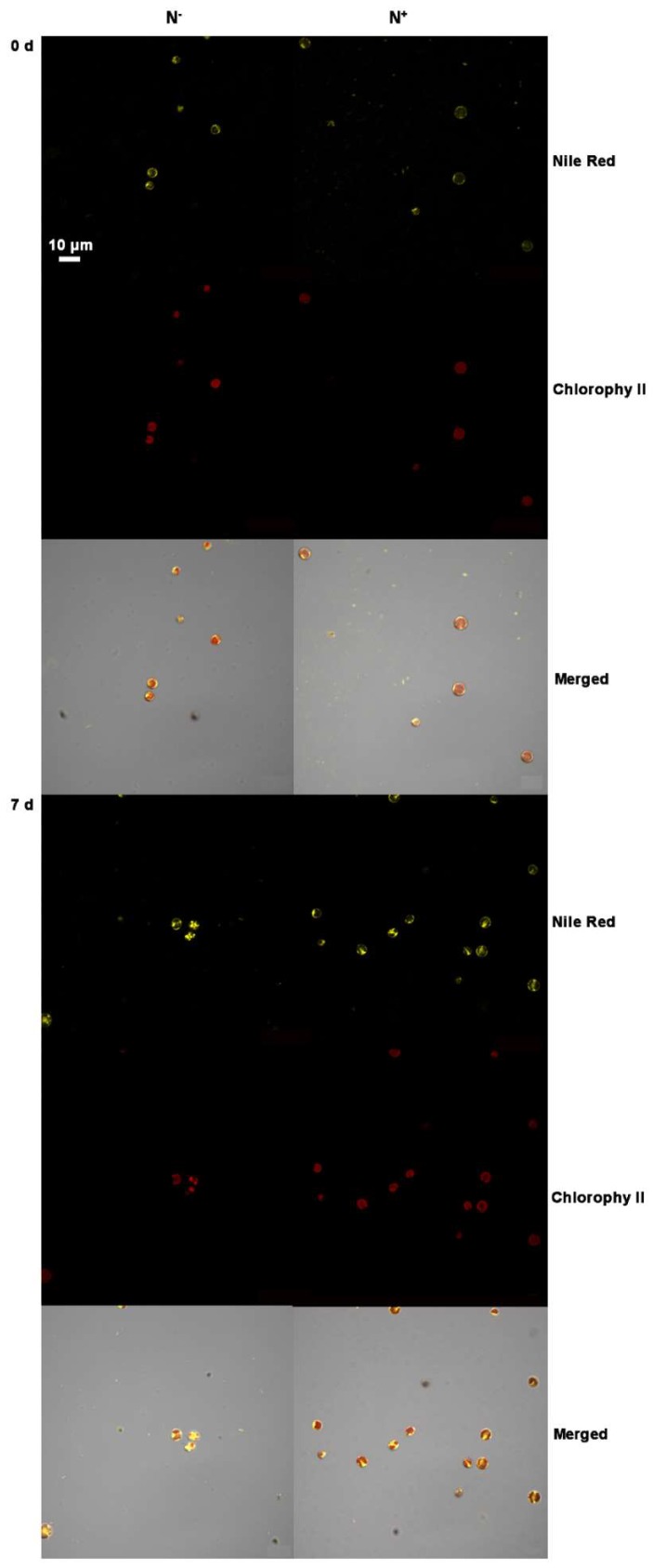
Representative confocal laser scanning micrographs of *Parachlorella kessleri* TY02 cells labeled with Nile Red. CLSM (confocal laser scanning microscopy) images of cells with NR fluorescence (yellow) and Chl autofluorescence (red) were recorded on the 0 day and 7th day under N^−^ and N^+^ culture media. All figures are representative of three replicated studies with similar findings. Scale bar: 10 μm.

Figure 6 shows the size and fluorescence intensity of lipid droplets in *P.kessleri* TY02 observed with confocal laser microscopy, under N^−^ and N^+^, at the beginning and on the 7th day, respectively. *P. kessleri* TY02 cells had some lipid droplets both under N^−^ and N^+^ at the beginning but their fluorescence intensities were weak. On the 7th day, the lipid droplets and fluorescence intensity increased significantly, especially under the N^−^ medium which elicited lipid droplets with a larger size and stronger fluorescence intensity.

### 3.4. Contents of Total Lipids

**Figure 7:**
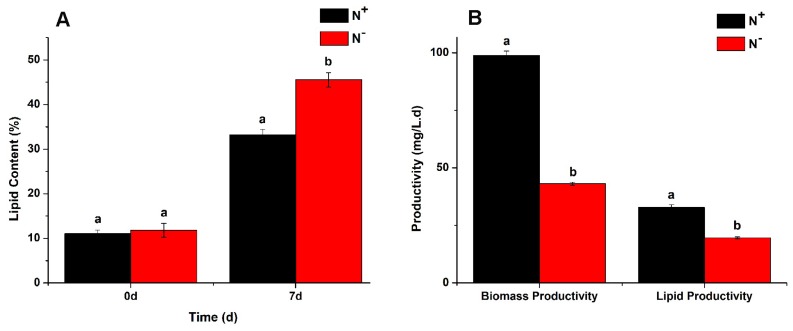
(**A**), Comparison of total lipid contents of *Parachlorella kessleri* TY02 under N^+^ and N^−^ culture media on the 0 day and 7th day, respectively. (**B**), Comparison of biomass productivity and lipid productivity of *P. kessleri* TY02 under N^+^ and N^−^ culture media on the 7th day. Data are shown as mean ± SE. n = 3. “aa” indicate non-significant differences (*p* > 0.05) and “ab” represent significant differences (*p* < 0.05).

As shown in Figure 7A, the total lipid contents of *P. kessleri* TY02 both under N^+^ and N^−^ at the beginning were 11.2% and 12.3%, respectively, and there was no significant difference between them (*p* > 0.05). When the algal cultivation broth was cultured to the 7th day, the contents increased to 33.2% and 45.5%, respectively, and the difference was statistically significant (*p* < 0.05). Besides, as shown in Figure 7B, the biomass productivity of *P. kessleri* TY02 under N^+^ and N^−^ culture media on the 7th day were 98.88 mg L^−1^d^−1^ and 43.12 mg L^−1^d^−1^, respectively. Similarly, the lipid productivity of *P. kessleri* TY02 under N^+^ and N^−^ culture media on the 7th day were 32.82 mg L^−1^d^−1^ and 19.61 mg L^−1^d^−1^, respectively. To sum up, although the nitrogen stress could promote the total lipid content, its lipid productivity was lower than the nitrogen-rich; the reason was that the biomass productivity of nitrogen-deficiency was relatively low.

### 3.5. Analysis of Fatty Acids

The fatty acid contents of *P. kessleri* TY02 under N^+^ and N^−^ are shown in Table 1. Fatty acids mainly including 7 categories were identified in *P. kessleri* TY02 under N^+^, and 8 categories under N. They were mainly composed of C16 and C18 fatty acids, and the content of C16 and C18 in the strain was up to 88.15% of *P. kessleri* TY02 under N^+^ and 92.43% of *P. kessleri* TY02 under N^−^. No long-chain fatty acid (more than C20) was determined. In addition to, the polyunsaturated fatty acids with more than four double bonds were also not found. The predominant fatty acids were C16:0 (N^+^: 22.32%, N^−^: 26.81%), C18:2 (N^+^: 13.9%, N^−^: 14.8%) and C18:3 (N^+^: 29.75%, N^−^: 30.2%) in *P. kessleri* TY02 both under N^+^ and N^−^. Besides, the SFA (saturated fatty acids) of N^−^ was more than N^+^, it was 28.07% under N^+^ and 33.13% under N^−^, similarly, the MUFA (monounsaturated fatty acids) of N^−^ was more than N^+^, it was 2.35% under N^+^ and 8.2% under N^−^ but the PUFA (polyunsaturated fatty acids) was different, it was 57.73% under N^+^ and 50.8% under N^−^, respectively. 

### 3.6. Total Protein Contents

**Figure 8:**
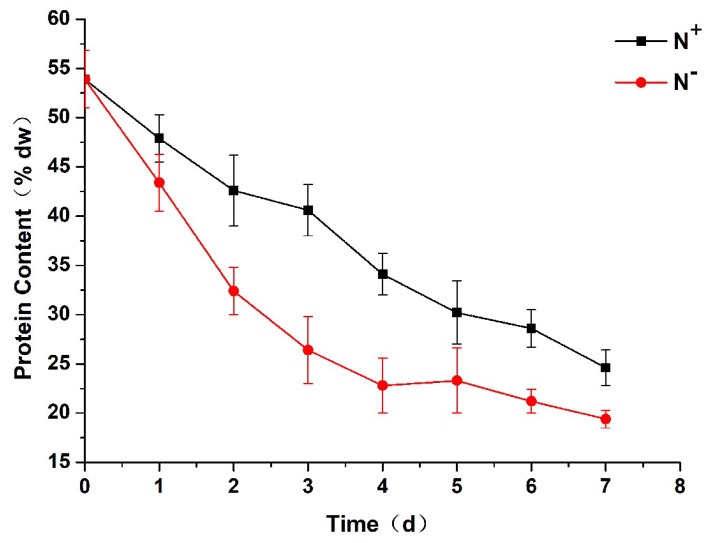
Comparison of total protein content of *Parachlorella kessleri* TY02 under N^+^ and N^−^ culture media. Data are shown as mean ± SE. *n* = 3.

As shown in Figure 8, the total protein contents of *P. kessleri* TY02 decreased both under N^−^ and N^+^ with the increase in culture time. However, compared with under N^+^, the total protein content decreased significantly under N^−^. At the beginning, the total protein content was 53.9% both under N^−^ and N^+^. On the 7th day, the total protein content under N^+^ was 24.6%, while it was only 19.4% under N^−^. This indicates that nitrogen stress could reduce protein synthesis.

### 3.7. Contents of Total Carbohydrates

**Figure 9:**
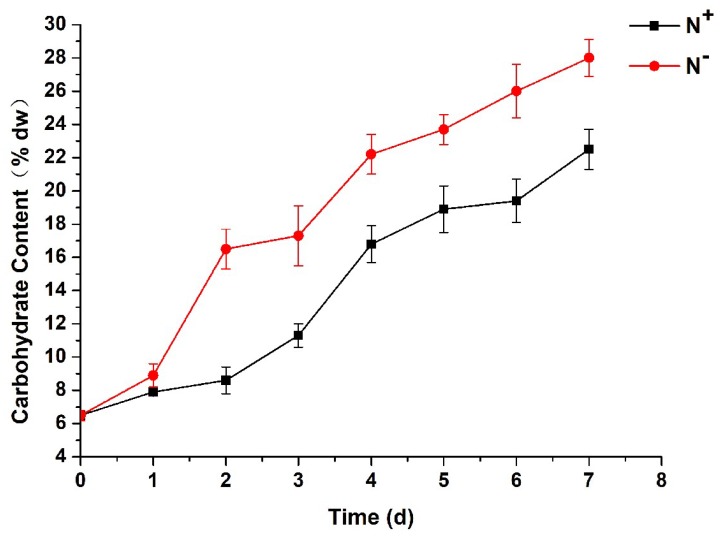
Comparison of total carbohydrate content of *Parachlorella kessleri* TY02 under N^+^ and N^−^ culture media. Data are shown as mean ± SE. *n* = 3.

As shown in Figure 9, the total carbohydrate contents of *P. kessleri* TY02 increased both under N^−^ and N^+^ with the increase in culture time. However, compared with under N^+^, the total carbohydrate content increased significantly under N^−^. At the beginning, the total carbohydrate content was about 6.5% both under N^−^ and N^+^. On the 7th day, the total carbohydrate content under N^+^ was 22.5%, while under N^−^ it was 28.1%. This further suggests that nitrogen stress could reduce protein synthesis, consequently causing an increase in carbohydrate contents. 

**Figure 10:**
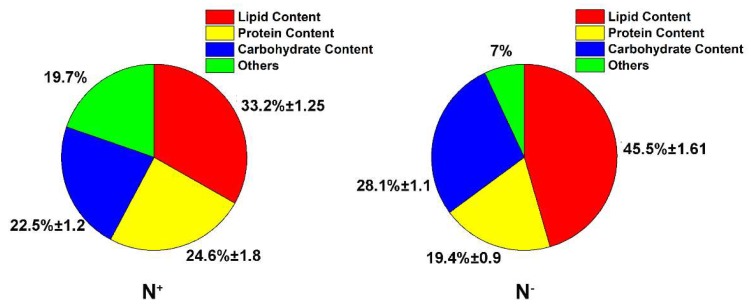
Comparison of biochemical composition (Lipid, Carbohydrate and Protein) of *Parachlorella kessleri* TY02 under N^+^ and N^−^ culture media on the 7th day. Data are shown as mean ± SE. *n* = 3.

Because biochemical composition (lipid, protein and carbohydrate) occupies an important place in biology, it is very important to know the content distribution in algae. As shown in Figure 10, the biochemical composition was made up of the bulk of the *P.kessleri* TY02 both under N^+^ and N^−^ conditions (more than 80% in N^+^ and over 90% in N^−^) on the 7th day, the lipid, protein and carbohydrate were 33.2%, 24.6% and 22.5% under N^+^ condition, and 45.4%, 19.4%, 28.1% under N^−^ condition, respectively. 

## 4. Discussion

Nitrogen is one of the most important nutrient factors that restricts the growth of microalgae and it is essential for microalgae to maintain normal metabolism. It was documented that many stress conditions could significantly affect algal growth and lipid accumulation, among which reducing nitrogen content is a relatively economical and easy-to-operate method [30]. The experimental results also proved this point. Nile Red (NR) is a lipid-soluble fluorescent probe that can be used for in *situ* determination in *vivo* and reacts with neutral lipids in cells [31]. In this study, the neutral lipid of *P. kessleri* TY02 was analyzed relative quantitative by adopted the NR combined with multiscan spectrum and laser confocal microscope, the approach had detection sensitivity, was fast, and had lower cost advantages. The fluorescence intensity after dyeing directly reflects the relative content of neutral lipids in *P. kessleri* TY02 cells and the two experimental results were consistent, indicating that nitrogen-deprivation was conducive to the accumulation of neutral lipids.

This study also measured and analyzed the experiments on the total lipid content and the fatty acids about *P.kessleri* TY02 under nitrogen stress. It was found that the total lipid content of *P.kessleri* TY02 was up to 45.5% when nitrogen was deficiency at the end of the culture stage. In addition, *P.kessleri* TY02 contained both saturated and unsaturated fatty acids, meanwhile, the main fatty acid compositions were hexadecanoic acid (C16:0), 9, 12-octadecadienoic acid (C18:2) and 9, 12, 15-octadecatrienoic acid (C18:3), of which the content of C16 and C18 were up to 88.15% under N^+^ and 92.43% under N^−^. According to the relevant references, C16 and C18 reached a high content of fatty acid, up to about 50–95%, 25–80% and 30–40% contents in Chlorophyta [32], Bacillariophyta and Dinophyta [33], respectively. In those microalgae, Chlorophyta had much more C16 and C18 than the other taxa. Besides, as FAME (fatty acid methyl esther) are relatively consistent with fatty acids present in the feedstock, this relation could be used to estimate the properties of biodiesel produced from the feedstock [34]. In this study, the SFA (saturated fatty acids) and MUFA (monounsaturated fatty acids) of N^−^ were both more than N^+^; the relevant research showed that the saturated fatty acids could offer oxidation resistance, which is beneficial to long-term storage. The monounsaturated fatty acids had low temperature stability; it would not be degraded due to the high oxidation, which is good for improving the low temperature performance of biodiesel [35]. Therefore, the *P. kessleri* TY02 under the nitrogen stress had the greatest application potential to produce biodiesel.

In addition, under restriction of nitrogen, the protein content was lower than that of the normal nitrogen group, which indicated that during the growth and metabolism of algae cells, some substances with higher nitrogen content (such as unnecessary proteins) were gradually decomposed and converted into storage substances with lower nitrogen content, such as the storage of oils and starches from intracellular, so the accumulation of the lipid (particularly the neutral lipid) and carbohydrate in the *P. kessleri* TY02 were promoted by nitrogen stress [36]. This indicated that protein synthesis decreased but carbohydrate and lipid content increased under the N^−^ condition, especially the accumulation of triglyceride (TAG) in algal cells, that is, algal cells distributed more carbon and energy into lipid synthesis [37,38,39,40].

What is more, a study by Msanne et al. [41] on *Chlamydomonas reinhardtii* showed that nitrogen stress could weaken algal photosynthesis, decrease photosynthetic activity, disrupt the metabolic balance of algal cells, block the transport of energy and nutrients required for proper growth, and gradually stop growth and division. These findings were consistent with the results obtained in the present study on *P. kessleri* TY02. Under nitrogen stress, the growth of algal cells was slowed down, the biomass was reduced, and the chlorophyll contents were also decreased significantly. The nitrogen deficiency negatively affected the synthesis of photosynthetic pigments in *P. kessleri* TY02. In addition, the ratio of Car to Chl (a+b) was negatively correlated with the level of nitrogen in the culture medium, which suggests that nitrogen stress was beneficial to the accumulation of carotenoids.

Although the biomass of *P. kessleri* TY02 under nitrogen stress was lower than in nitrogen-rich conditions, which also leads to the low lipid productivity, the total lipid content and neutral lipid content under the nitrogen stress were higher than in the nitrogen-rich conditions. Besides, in the analysis of fatty acids, the SFA and MUFA which were suitable for producing biodiesel, had higher contents under nitrogen stress than in the nitrogen-rich conditions. Of course, we should undertake further study on how to improve biomass on the basis of maintaining high total lipid content and favorable fatty acid content. From now on, it is necessary to further optimize the *P. kessleri* TY02 under a nitrogen-deficiency culture, for example, *P. kessleri* TY02 can be cultivated by different culture stages; at first, the microalgae could culture in the nitrogen-rich medium to ensure high biomass, then be cultured in another stage (nitrogen-deficiency), or the *P. kessleri* TY02 can be cultivated in the heterotrophic culture under the nitrogen-deficiency medium [42]. So, a study on the physiological mechanism of *P. kessleri* TY02 under the nitrogen deficiency condition is very necessary and important, and provides a certain foundation for the following research.

## 5. Conclusions

The effects of nitrogen stress on the growth, photosynthetic pigments, lipid content, protein content and carbohydrate contents of *P. kessleri* TY02 were studied. The results showed that, under nitrogen stress, the algal strain grew slowly, chlorophyll and total protein contents decreased, and was lower than the nitrogen-rich, but the total carbohydrate and total lipid (especially neutral lipid) contents increased significantly. In addition, under the nitrogen stress, it had more SFA and MUFA than in nitrogen-rich conditions, which showed that it is much more suitable for producing biodiesel. What is more, the present results suggest that, under nitrogen stress, most of the carbon is allocated to the synthesis of lipids and carbohydrates. In short, reducing nitrogen content is a relatively economical and easy-to-operate method of promoting lipid accumulation in microalgae.

## Figures and Tables

**Table 1:** Fatty acids profiles (percentage of total fatty acids) in *Parachlorella kessleri* TY02 under N^+^ and N^−^ culture conditions.

Fatty Acids	Nitrogen-Rich (N^+^)	Nitrogen-Deficient (N^−^)
C16: 0	22.32 ± 0.38	26.81 ± 0.31
C16: 1	2.35 ± 0.1	4.15 ± 0.12
C16: 2	5.27 ± 0.08	2.1 ± 0.11
C16: 3	8.81 ± 0.21	3.7 ± 0.13
C18: 0	5.75 ± 0.12	6.32 ± 0.1
C18: 1	—	4.05 ± 0.02
C18: 2	13.9 ± 0.23	14.8 ± 0.41
C18: 3	29.75 ± 0.29	30.2 ± 0.48
C16 and C18	88.15	92.43
Others	11.85	7.57
SFA (Saturated Fatty Acids)	28.07	33.13
MUFA (Monounsaturated Fatty Acids)	2.35	8.2
PUFA (Polyunsaturated Fatty Acids)	57.73	50.8

Values are means ± standard deviations of triplicates.
